# Enzymatic properties of *Thermoanaerobacterium thermosaccharolyticum* β-glucosidase fused to *Clostridium cellulovorans* cellulose binding domain and its application in hydrolysis of microcrystalline cellulose

**DOI:** 10.1186/1472-6750-13-101

**Published:** 2013-11-14

**Authors:** Linguo Zhao, Qian Pang, Jingcong Xie, Jianjun Pei, Fei Wang, Song Fan

**Affiliations:** 1 College of Chemical Engineering, Nanjing Forestry University, Nanjing 210037, China; 2 Jiangsu key Lab of Biomass Based Green Fuels and Chemicals, Nanjing, China

**Keywords:** Glucose-tolerant β-glucosidase, Cellulose binding domain (CBD), Fusion protein, Immobilization

## Abstract

**Background:**

The complete degradation of the cellulose requires the synergistic action of endo-β-glucanase, exo-β-glucanase, and β-glucosidase. But endo-β-glucanase and exo-β-glucanase can be recovered by solid–liquid separation in cellulose hydrolysis by their cellulose binding domain (CBD), however, the β-glucosidases cannot be recovered because of most β-glucosidases without the CBD, so additional β-glucosidases are necessary for the next cellulose degradation. This will increase the cost of cellulose degradation.

**Results:**

The glucose-tolerant β-glucosidase (BGL) from *Thermoanaerobacterium thermosaccharolyticum* DSM 571 was fused with cellulose binding domain (CBD) of *Clostridium cellulovorans* cellulosome anchoring protein by a peptide linker. The fusion enzyme (BGL-CBD) gene was overexpressed in *Escherichia coli* with the maximum β-glucosidase activity of 17 U/mL. Recombinant BGL-CBD was purified by heat treatment and following by Ni-NTA affinity. The enzymatic characteristics of the BGL-CBD showed optimal activities at pH 6.0 and 65°C. The fusion of CBD structure enhanced the hydrolytic efficiency of the BGL-CBD against cellobiose, which displayed a 6-fold increase in *V*_*max*_/*K*_*m*_ in comparison with the BGL. A gram of cellulose was found to absorb 643 U of the fusion enzyme (BGL-CBD) in pH 6.0 at 50°C for 25 min with a high immobilization efficiency of 90%. Using the BGL-CBD as the catalyst, the yield of glucose reached a maximum of 90% from 100 g/L cellobiose and the BGL-CBD could retain over 85% activity after five batches with the yield of glucose all above 70%. The performance of the BGL-CBD on microcrystalline cellulose was also studied. The yield of the glucose was increased from 47% to 58% by adding the BGL-CBD to the cellulase, instead of adding the Novozyme 188.

**Conclusions:**

The hydrolytic activity of BGL-CBD is greater than that of the Novozyme 188 in cellulose degradation. The article provides a prospect to decrease significantly the operational cost of the hydrolysis process.

## Background

Cellulosic biomass is the most abundant renewable resource on earth, whose natural degradation represents an important part of the carbon cycle within the biosphere [1]. The complete degradation of the cellulose requires the synergistic action of endo-β-glucanase (EC 3.2.1.4), exo-β-glucanase (EC 3.2.1.91), and β-glucosidase (EC 3.2.1.21) [2,3]. One of the limiting steps in the enzymatic saccharification of cellulosic material is the conversion of short-chain oligosaccharides and cellobiose, which was resulted from the synergistic action of endogucanases and cellobiohydrolases, to glucose, a reaction catalyzed by β-glucosidases [4]. It is well established that cellobiose inhibits the activities of most cellobiohydrolases and endoglucanses [5]. β-glucosidases reduce cellobiose inhibition by hydrolyzing the disaccharide to glucose, thus allowing the cellulolytic enzymes to function more efficiently. It has been shown that the supplementation of commercially produced cellulases from fungal sources such as *Trichoderma reesei* with β-glucosidase produced by *Aspergillus niger* increases the rate and extent of glucose production [5-7].

Endo-β-glucanase and exo-β-glucanase can be recovered by solid–liquid separation in cellulose hydrolysis by their cellulose binding domain (CBD), but the β-glucosidases cannot be recovered because of most β-glucosidases without the CBD, so additional β-glucosidases are necessary for the next cellulose degradation. This will increase the cost of cellulose degradation. Moreover, it has been suggested that the CBD enhances the enzymatic activity of cellulolytic enzymes simply by reducing the dilution effect of the enzyme at the substrate surface, by promoting the solubilization of single glucan chains of the cellulose surface, or by loosening individual cellulose chains from the cellulose surface prior to its actual hydrolysis [8,9]. Thus, it would be interesting to study the effects of the CBD on the cellulose degradation of β-glucosidases. In nature, a β-glucosidase with an N-terminal CBD from *Phanerochaete chrysosporium* has been purified and characterized [10]. Further, Sarath reported the effect of a fungal CBD on the enzymatic characteristics of the β-glucosidase from *Saccharomycopsis fibuligera*. The fusion enzyme displayed a 2–4 fold increasing in their hydrolytic activity toward cellulosic substrates [11]. In the present study, we successfully over-expressed the β-glucosidase (BGL) gene from *T. thermosaccharolyticum* DSM 571 in *E. coli*. As compared on the enzyme properties, the BGL showed higher tolerant to glucose and cellobiose, more efficient in hydrolysis of cellobiose, more thermal stability than β-glucosidases from other microorganisms [12].

In this work, the glucose-tolerant β-glucosidase (BGL) from *T. thermosaccharolyticum* DSM 571 was fused with cellulose binding domain (CBD) of *Clostridium cellulovorans* cellulosome anchoring protein, the biochemical characterization and cellulose binding property of BGL-CBD were determined, and its application in hydrolysis of cellulose was evaluated.

## Methods

### Bacterial strains, plasmids, growth media

*Escherichia coli* JM109 and JM109(DE3) was grown at 37°C in Luria-Bertani medium (LB) and supplemented with ampicillin when required. The expression vectors pET-20b (Novagen) were employed as cloning vector and expression vector. The plasmid pET-20-BGLII was reserved by our laboratory [12]. The genomic DNA of *Clostridium cellulovorans* 743B was purchased from DSMZ (http://www.dsmz.de).

### DNA Manipulation

DNA was manipulated by standard procedures [13]. QIAGEN Plasmid Kit and QIAGEN MinElute Gel Extraction Kit (Qiagen, USA) were employed for the purification of plasmids and PCR products. DNA restriction and modification enzymes were purchased form TaKaRa (Dalian, China). DNA transformation was performed by electroporation using GenePulser (Bio-Rad, USA).

### Plasmid constructions

The β-glucosidase gene *bgl* was amplified from plasmid pET-20-BGLII by PCR using primers bgl-1: CCCCATATGAGCGATTTTAACAAAGAT and bgl-2: CCCGGATCCAATGGTCCTAGTGGAAATAAG. The underlined sequences represented the restriction enzyme sites. The PCR products were digested with *Nde* I and *BamH* I, and inserted into pET-20b at *Nde* I and *BamH* I sites, yielding the plasmid pET-BGL.

The CBD encoding gene fragment was amplified from genomic DNA of *Clostridium cellulovorans* by PCR using primers CBD-1: CCCGGATCCATGTCAGTTGAATTTTACAA and CBD-2: CCCCTCGAGTGGTGCTGTACCAAGAACT. The underlined sequences represented the restriction enzyme sites. The PCR products were digested with *BamH* I and *Xho* I, and inserted into pET-BGL at *BamH* I and *Xho* I sites, yielding the plamid pET-BGL-CBD.

The CBD encoding gene with a peptide linker fragment was amplified from genomic DNA of *C. cellulovorans* by PCR using primers CBD-3: CCCGGATCC*CCACCACCA*ATGTCAGTTGAATTTTACAA and CBD-2. The underlined sequences represented the restriction enzyme sites, and the italic sequences represented the linker peptide. The PCR products were digested with *BamH* I and *Xho* I, and inserted into pET-BGL at *BamH* I and *Xho* I sites, yielding the plamid pET-BGL-Linker-CBD.

### Expression and purification of the fusion enzyme

Plasmids pET-BGL-CBD and pET-BGL-Linker-CBD were transformed into *E. coli* JM109(DE3), and induced to expression recombinant BGL-CBD by adding isopropyl-β-D-thiogalactopyranoside (IPTG) to final concentration of 0.5 mM at OD_600_ about 0.7, and incubated further at 30°C for about 6 h.

The recombinant cells (200 mL) carrying pET-BGL-CBD or pET-BGL-Linker-CBD were harvested by centrifugation at 5,000 g for 10 min at 4°C, and washed twice with distilled water, resuspended in 50 mL of 5 mM imidazole, 0.5 mM NaCl, and 20 mM Tris–HCl buffer (pH 7.9), and French-pressured for three times. The cell extracts were heat treated (60°C, 30 min), and then cooled in an ice bath, and centrifuged (20,000 g, 4°C, 30 min). Afer heat treatment (60°C, 30 min), the resulting supernatants were loaded on to an immobilized metal affinity column (Novagen, USA), and eluded with 1 M imidazole, 0.5 M NaCl, and 20 mM Tris–HCl buffer (pH 7.9). Protein was examined by SDS-PAGE [14], and the protein bands were analyzed by density scanning with an image analysis system (Bio-Rad, USA). Protein concentration was determined by the Bradford method using BSA as a standard.

### Determination of the fusion enzyme activities and properties

The reaction mixture, containing 50 mM citrate buffer (pH 6.0), 1 mM p-nitrophenyl-β-D-glucopyranoside, and certain amount of β-glucosidase in 0.2 mL, was incubated for 5 min at 65°C. The reaction was stopped by the addition 1 mL of 1 M Na_2_CO_3_. The absorbance of the mixture was measured at 405 nm. One unit of enzyme activity was defined as the amount of enzyme necessary to liberate 1 μmol of *p*NP per min under the assay conditions.

The optimum pH for the fusion enzyme was determined by incubation at 65°C for 5 min in the 50 mM citrate buffer from pH 4.0 to 7.5. The optimum temperature for the fusion enzyme was determined by standard assay ranging from 45 to 80°C in the 50 mM citrate buffer, pH 6.0. The results were expressed as percentages of the activity obtained at either the optimum pH or the optimum temperature.

The pH stability of the fusion enzyme was determined by measuring the remaining activity after incubating the fusion enzyme at 50°C for 1 h in the 50 mM citrate buffer from pH 4.5 to 7.5. To determine the effect of temperature on the stability of the fusion enzyme, the fusion enzyme (0.1 μg) in the 50 mM citrate buffer (pH 6.0) was pre-incubated for 1 h at 40°C, 45°C, 50°C, 55°C, 60°C, 65°C, and 70°C in the absence of the substrate. The activity of the enzyme without pre-incubation was defined as 100%.

Kinetic constant of the fusion enzyme was determined by measuring the initial rates at various p-nitrophenyl-β-D-glucopyranoside concentrations (0.2, 0.4, 0.6, 0.8, 1, 2, and 4.0 mM) or various cellobiose concentration (2, 4, 6, 8, 10, 12, 14, and 16 mM) under standard reaction conditions. The *K*_*i*_ value of glucose was defined as amount of glucose required for inhibiting 50% of the β-glucosidase activity and was given as the averages of three separate experiments performed in duplicate.

### Adsorption assays

The fusion enzyme (12 U, 10 mL, pH 6.0) was mixed with the Avicel PH101 (Sigma, USA) at 50°C, 100 rpm. The influence of process parameters on absorption were determined by varying adsorption time, NaCl concentration, and pH value. The relevant samples were centrifuged to discard the supernatant fluids. The resulting pellets were washed twice by 50 mM citrate buffer (pH 6.0) and assayed for β-glucosidase activity adsorbed on the cellulose.

### Adsorption isotherm measurements

To estimate the binding capacity of BGL-CBD attached to the Avicel, adsorption isotherm measurements was taken. A sequence of test tubes containing 10 mL of various BGL-CBD concentrations (0.005 mg/mL to 0.02 mg/mL). To each tube, 0.016 g of the Avicel was added and incubated for 25 min at 50°C, 100 rpm, pH 6.0. The suspension was centrifuged at 10,000 g for 10 min. The concentrations of BGL-CBD were determined by absorbance at 280 nM and assay of β-glucosidase activity.

### Analysis of cellobiose and reusability assay

The cellobiose was treated with the fusion enzyme, and the degradation was subjected to analysis of glucose assay kit (Dingguo, China). The reaction mixture (400 μL) contained 290 mM cellobiose, and BGL-CBD (0.1 μg/μL) in 50 mM citrate buffer (pH 6.0). The reaction was performed for various times at 55°C, and stopped by heating for 5 min in a boiling water bath.

The fusion enzyme was incubated with cellobiose for various times at 50°C. Then the reactions were mixed with cellulose for 25 min at 50°C and were centrifuged. The resulting supernatants were collected for sugar analysis. The pellets were washed twice and supplement with fresh cellobiose to initiate another cycle. Five batches were performed according to the same procedure. The activity of the enzyme in the first run was defined as 100%.

### Avicel PH101 hydrolysis using cellulase in combination with BGL-CBD and Novozyme 188

Cellulose degradation experiments with 5% (w/v) Avicel PH101 were performed in a 50 mM citrate buffer at 180 rpm, pH 5.2 and 50°C. The total working volume was 10 in 50 mL triangular flasks. The two commercial enzyme solutions, Celluclast 1.5 L (115 filter paper units (FPU)/mL) and Novozyme 188 (328 glucosidase units (CBU)/mL) were obtained from Sigma-Aldrich. The enzyme dosage was 15 FPU and 30 CBU/g glucan, respectively, for celluclast 1.5 L and Novozyme 188 or BGL-CBD. After 12 h of incubation, the enzymes were recovered using centrifugation 15 min at 5, 000 g. Subsequently, they were used for a second hydrolysis cycle using the same conditions described above without adding enzymes. This was performed in total of three campaigns. The glucose was subjected to analysis of glucose assay kit (Dingguo, China). The glucose yield was calculated according to the following equations [15]: Glucose yield (%) = 0.9*100*glucose (g)/initial cellulose or cellobiose (g).

## Results and discussion

### Gene cloning and production of BGL-CBD fusion

**Figure 1:**
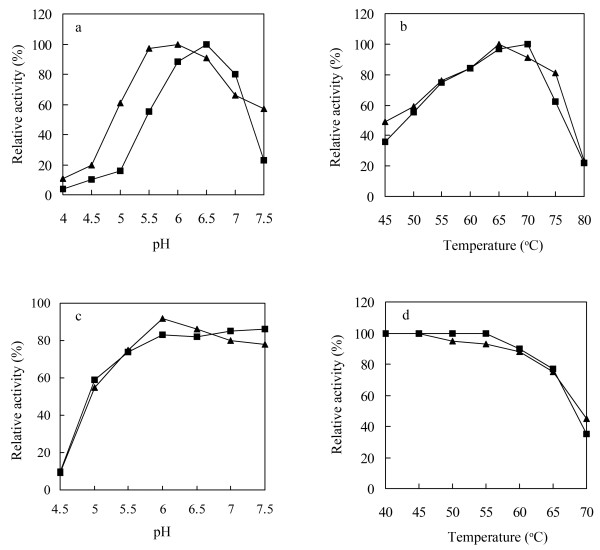
**The effects of pH and temperature on the activity and stability of the BGL-CBD and BGL. a** Effect of pH on the activity. **b** Effect of temperature on the activity. **c** The pH stability of the enzymes. **d** The thermostability of the enzymes. The filled triangle represented BGL-CBD and filled square represented BGL. The initial activity was defined as 100%.

For the fusion of BGL with CBD, the plasmids pET-BGL-CBD and pET-BGL-Linker-CBD were successfully constructed by linking the *T. thermosaccharolyticum* DSM 571 glucose-tolerant β-glucosidase gene *bgl*[12] to the gene fragment encoding CBD from *C. cellulovorans* cellulosome anchoring protein with or without a flexible peptide Linker (PPP). The BGL-CBD without a flexible peptide linker was identified with worse thermostability than the BGL, and thermostability assays indicated that its residual activity was less than 10% after being incubated at 50°C for 20 min (data not shown). A key consideration in construction of fusion enzymes is the preservation or improvement of the protein and enzymatic characteristics of the individual components. Simple head-to-tail in-frame fusions of two proteins often result in non-functional proteins due to either incorrect folding or restrained ability to interact with other protein subunits [16]. To increase the thermostability of the BGL-CBD, a flexible peptide linker containing (ProProPro) was inserted in the middle of the BGL-CBD, which often conferred the necessary flexibility for the individual proteins to maintain their functionalities. As expected, the BGL-CBD with a flexible peptide linker showed the good thermostability (Figure 1d).

### Characterization of recombinant BGL-CBD fusion

**Figure 2:**
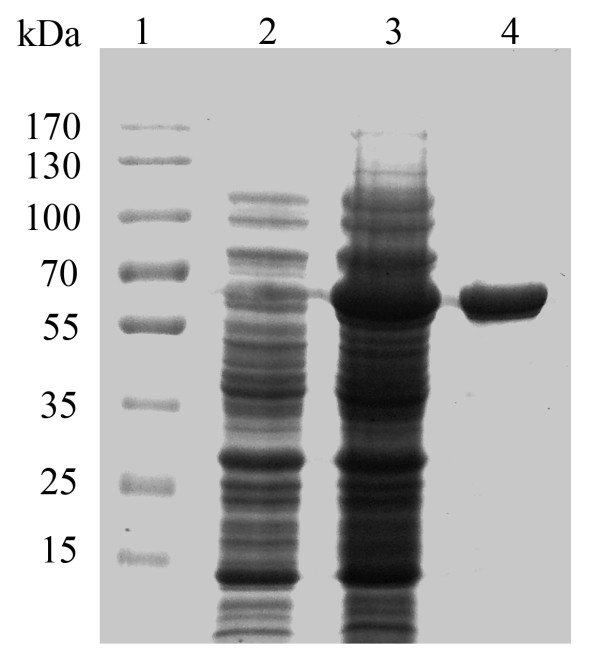
**SDS-PAGE analysis of recombinant BGL-CBD in *E. coli *JM109(DE3).** Lane M: protein marker, lane 1: cell-free extract of JM109(DE3) harboring pET-20b, lane 2: cell-free extract of JM109(DE3) harboring pET-BGL-Linker-CBD, lane 3: purified BGL-CBD (5 μg).

**Figure 3:**
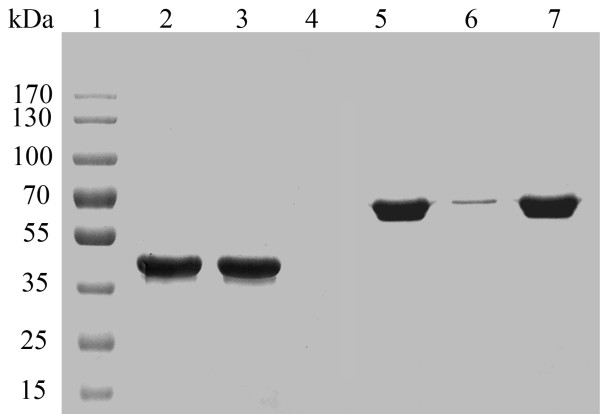
**Adsorption of BGL-CBD and BGL to cellulose.** Lane 1: protein marker, lane 2: purified BGL, lane 3: the supernatant of BGL binding to cellulose, lane 4: cellulose binding BGL, lane 5: purified BGL-CBD, lane 6: the supernatant of BGL-CBD binging to cellulose, lane 7: cellulose binding BGL-CBD.

The fusion enzymes (BGL-CBD) were expressed in *E. coli* and purified to electrophoretic homogeneity by heat treatment followed by Ni-NTA affinity (Figure 2). The β-glucosidase activity expression from pET-BGL-Linker-CBD was 17 U/mL, which was about 2.2 times higher than the expressed from pET-20-BGL [12]. The molecular weight of the fusion enzyme was estimated to be about 69 kDa in comparison to 52 kDa of the native enzyme (BGL) (Figure 3). The fusion enzymes were purified by 3.8-fold, and its specific activity reached 80.5 U/mg (Table 1).

**Table 1:** **Purification of BGL-CBD from *E. coli *harboring pET-BGL-Linker-CBD**

**Steps**	**Total protein (mg)**	**Total activity (U)**	**Specific activity (U/mg)**	**Yield (%)**
Cell extract	158	3357	21.2	100
Heat treatment	89	2954	33.2	88%
Ni-NTA affinity	32	2578	80.5	77%

The biochemical properties were investigated by using the purified recombinant enzyme. The data illustrates a diverse trend of hydrolytic pattern between the native enzyme BGL and the fusion enzyme BGL-CBD. The BGL, tested for activity showed a quick increasing in activity through pH 5.0-6.0 up to the highest activity at pH 6.5. The BGL-CBD displayed the optimal activity at pH 6.0 (Figure 1a). It is important for the β-glucosidase to remain active at low pH, because the optimal pH of the commercial cellulase from *T. reesei* was 4.8 [17]. The addition of the β-glucosidase must be right for the commercial cellulase. The activity of BGL was only 16% of the maximum activity at the pH 5.0, while the activity of BGL-CBD was higher than 60% of the maximum activity at the pH 5.0, so the BGL-CBD is more adaptable in the application of cellulose hydrolysis than BGL. The optimal temperature for the activity of the BGL-CBD was 65°C, slightly lower than that of the BGL (70°C) (Figure 1b), while the pH and thermal stabilities of the BGL-CBD were similar to those of the BGL (Figure 1c, d). The BGL-CBD residual activity was more than 90% after being incubated at 60°C for 1 h. The stability of enzyme during catalysis reaction and longtime storage is an important factor from the viewpoint of industrial application [18].

**Figure 4:**
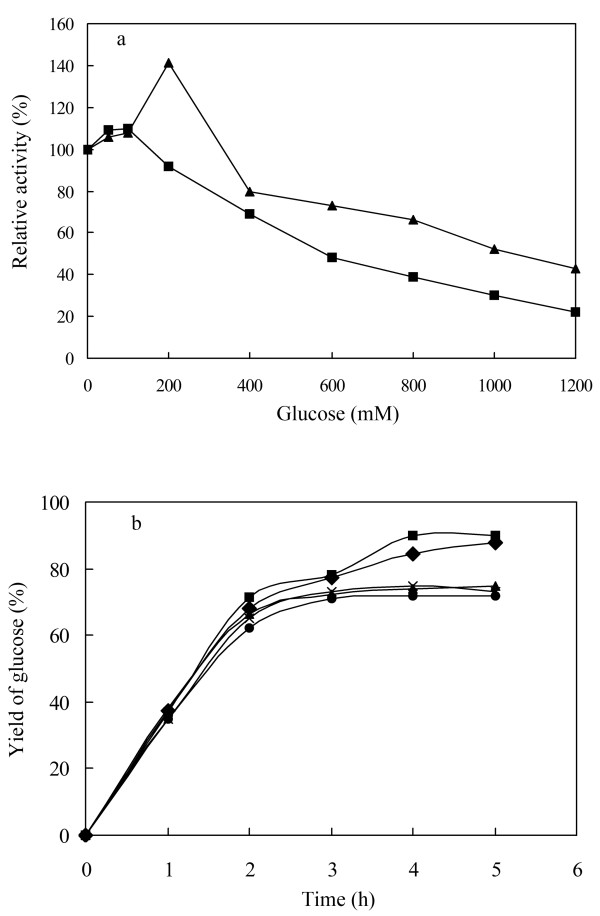
**The effects of glucose on BGL-CBD activity and analysis of cellobiose hydrolysed by BGL-CBD. a** Influence of glucose on enzyme activity with p-nitrophenyl-β-D-glucopyranoside as the substrate. The filled triangle represented BGL-CBD and filled square represented BGL. **b** Analysis of cellobiose hydrolysed by BGL-CBD. The filled square represented the first cycle, the filled diamond represented the second cycle, the filled triangle represented third cycle, the letter x represented the fourth cycle, and the filled circle represented fifth cycle.

It has been suggested that the CBD enhances the enzymatic activity of cellulolytic enzymes simply by reducing the dilution effect of the enzyme at the substrate surface [19], by promoting the solubilization of single glucan chains off the cellulose surface [20], or by loosening individual cellulose chains from the cellulose surface prior to its actual hydrolysis [8]. The Michaelis-Menten constants for the BGL-CBD and the BGL are listed in Table 2. The *V*_*max*_/*K*_*m*_ value of the BGL-CBD for cellobiose was 90 U/mg_*_mM, which was six times higher than that of BGL. Furthermore, the activity of BGL-CBD was enhanced by the concentration of glucose below 200 mM, and the enzyme activity was increased by 140% when adding 200 mM glucose into reaction mixtures. With glucose further increasing, the activity of BGL-CBD was gradually inhibited, with a *K*_*i*_ of 1200 mM glucose (Figure 4a). The BGL-CBD yielded approximately two times higher *K*_*i*_ values for glucose than the BGL. These results indicated that the CBD coupled to a non-cellulolytic β-glucosidase enzyme modulates its ability to hydrolyze cellobiose and tolerate glucose.

**Table 2:** Michaelis constants for enzymes

**Enzymes**	**Substrate**	***K***_***m ***_**(mM)**	***V***_***max ***_**(U/mg)**
BGL-CBD	pNPG	0.25	134
	Cellobiose	2.62	236
BGL	pNPG	0.62	64
	Cellobiose	7.9	120

### Immobilization of BGL-CBD onto microcrystalline cellulose

**Figure 5:**
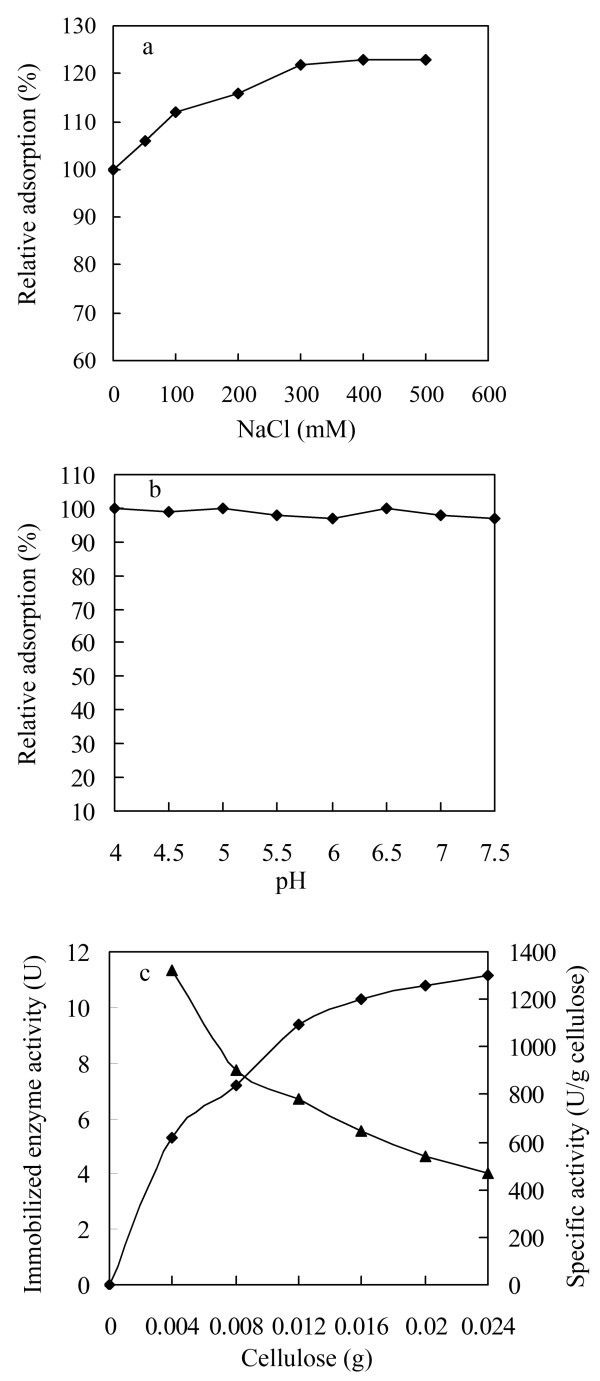
**Effects of environmental factors on the adsorption efficiency of BGL-CBD.** Effects of NaCl concentration **(a)**, pH **(b)** and cellulose amounts **(c)** on the adsorption of the BGL-CBD protein on cellulose.

The purified BGL-CBD enzyme was mixed with microcrystalline cellulose in the quantitative experiments. The adsorption of BGL-CBD on cellulose reached plateau in 25 min (data not shown). It was reported that binding forces between CBD and cellulose were attributed mainly to hydrophobic interactions [21]. The adsorption efficiency might be influenced by a series of environmental factors, such as temperature, salt concentration, and solution pH. The binding was obviously increased by 120% with increasing NaCl concentrations from 0 to 300 mM, as shown in Figure 5a. The rise of binding became slight when the NaCl concentration was larger than 300 mM. Even the adsorption increased by no more than 2%, when continuously increasing NaCl concentrations was up to 500 mM. The effect of solution pH on the adsorption was not so significant (Figure 5b).

**Figure 6:**
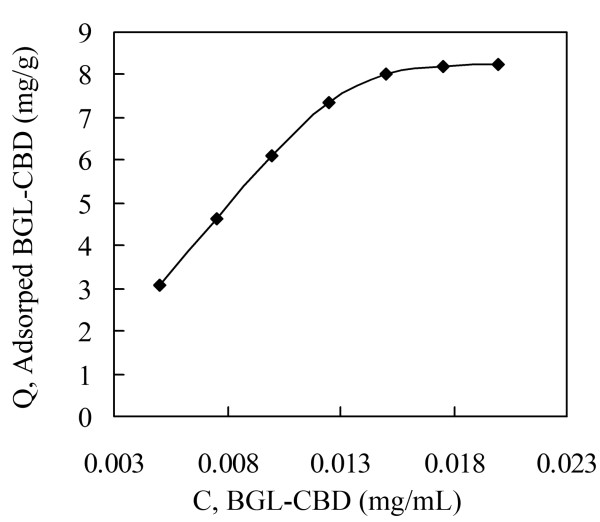
**Adsorption isotherm of BGL-CBD on Avicel PH101 and its Langmuir model.** C represented initial concentration of the fusion enzyme (BGL-CBD), Q represented the fusion enzyme adsorpted by Avicel PH101.

To investigate the immobilization efficiency, various amount of cellulose were mixed with 12 U of purified BGL-CBD. As shown in Figure 5c, the total immobilized enzyme activity increased notably with increasing cellulose amounts up to 0.016 g, and then the rise became slight and nearly reached a plateau. In contrast, the specific activity of enzyme immobilized on per gram of the matrix was consistently reduced with the increase of cellulose amounts. Considering the industrial cost, a gram of cellulose was found to absorb 643 U of enzyme (BGL-CBD) in pH 6.0 at 50°C for 25 min with a high immobilization efficiency of 90%. On the contrary, the cellulose is hard to absorb the BGL in the same conditions (Figure 3). The purified enzyme was used to evaluated the adsorption isotherm of BGL-CBD on Avicel. The amount of equilibrium adsorption of BGL-CBD on a constant amount of Avicel increased gradually with the increase of BGL-CBD concentration in the solution. Figure 6 shows that the isothem of adsorption of the fusion enzyme on Avicel is well coincident with that of Langmuir isotherm model, and in condition of 50°C and pH 6.0, the equilibrium adsorbing capacity is 8.3 mg/g (668 U/g), which is close to the exprerimental value.

### Analysis of cellobiose degradation and reusability

Production of glucose from 290 mM cellobiose (10%) by the BGL-CBD was examined. As shown in Figure 4b, the hydrolysis rate of cellobiose increased remarkably with increasing time up to 2 h, and then the rise became slight. The hydrolysis rate of cellobiose reached 90% in 4 h. The hydrolytic activity of the BGL-CBD was greater than that of the BGL [12], because the *V*_*max*_/*K*_*m*_ value of the BGL-CBD for cellobiose was six times higher than that of BGL.

The operational stability of the BGL-CBD was evaluated through the repeated process. The BGL-CBD enzyme retained over 85% of its initial activity after successive utilization for 5 batches. The hydrolysis rates of cellobiose were all above 70% for each batch (Figure 4b). These results suggested that the BGL-CBD is quite stable in the applications. This high operational stability could significantly reduce the operation cost in industrial application.

### Application in producing glucose from cellulose (Avicel PH101)

**Figure 7:**
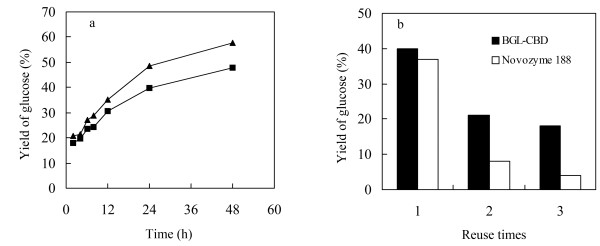
**Analysis of Avicel PH101 hydrolysis. a**. Time courses of Avicel PH101 hydrolysis by cellulase and BGL-CBD or cellulase and the Novozyme 188. The filled triangle represented BGL-CBD and filled square represented BGL. **b**. The yields of glucose in different cycles.

To order to study the effect of the BGL-CBD on more complex substrate, Avicel PH101 was used. The BGL-CBD or Novozyme 188 was combined with the cellulase (Celluclast 1.5 L) during the hydrolysis trials. Figure 7a showed that the yields of glucose using the cellulase with Novozyme 188 and the cellulase with the BGL-CBD were 47% and 58%, respectively. The yields of glucose increased 23.4% with the BGL-CBD in substitution for the Novozyme 188.

Moreover, the endo-β-glucanase and exo-β-glucanase from *T. reesei* have a conserved tripartite structure with a large catalytic core domain linked by an O-glucosylated peptide to a cellulose-binding domain (CBD), which is required for interaction with crystalline cellulose [8,9]. Thus, endo-β-glucanases and exo-β-glucanases can also be absorbed by the residual cellulose, and be reused for the next process. Thus, the cellulase with Novozyme 188 and the cellulase with the BGL-CBD were recovered by centrifugation 15 min at 5, 000 g, washed, and then used for a new hydrolysis cycle with the fresh substrate, respectively. It can be observed that after the first cycle, the yields of glucose were above 18% (BGL-CBD), which was higher than the Novozyme 188. But the yields of glucose were only half of the yields of glucose in the first cycle (Figure 7b). The loss in the yields of glucose could be due to the deactivation of enzymes during each hydrolysis cycle or to loss of immobilized enzyme cellulose during the separation. But no matter what, the results confirm that the BGL-CBD shows promise for cellulose hydrolysis.

## Conclusions

This work provided an efficient method for cellulose hydrolysis by the cellulose-binding fusion β-glucosidase. The BGL-CBD displayed a 6-fold increase in *V*_*max*_/*K*_*m*_ for cellobiose in comparison with the BGL. The BGL-CBD immobilized orientedly on to cellulose with high efficiency (90%). Using the BGL-CBD as the catalyst, the yield of glucose reached a maximum of 90% from 100 g/L cellobiose (pH 6.0) at 50°C for 4 h. The BGL-CBD could retain over 85% activity after five batches with the glucose yields all above 70%. Moreover, the hydrolytic activity of BGL-CBD is greater than that of the Novozyme 188 in cellulose degradation.

## Competing interests

The authors declare that they have no competing interests.

## Authors’ contributions

LZ and QP carried out the cloning and over-expression and drafted the manuscript. JX and SF helped to purified and characterized the BGL-CBD. FW reviewed and commented the manuscript. JP directed the over-all study and drafted the manuscript. All authors read and approved the final manuscript.
